# Supplementary material for: Hepatitis C virus seroprevalence among patients enrolled at the opioid substitution therapy center in Bihar: A cross-sectional study
Source: PLoS One. 2023 Jun 15;18(6):e0287333. doi: 10.1371/journal.pone.0287333 (PMC10270565; doi:10.1371/journal.pone.0287333)
Supplement: Attachment — Submitted filename: 2023.pdf [file pone.0287333.s001.pdf]

Review of PONE-D-22-32802, Panday et al.

This manuscript reports results from testing for hepatitis C virus (HCV) infection among patients at an opioid treatment program (OTP) in Bihar, in northeast India. Information on the small sample of patients (N=214), primarily from data collected at program entry (with the exception of mortality data) cover testing for infectious disease including HIV, HCV and other hepatitis viruses, sociodemographic data, drug use history including current and past injection. The sample is described as composed entirely of people who have or continue to inject drugs (PWID). There remain multiple issues that need to be clarified for this report to offer a compelling description of the sample and the results. There are also a large substantial number of typographical, grammatical, and semantical errors that need to be addressed.

First, I will list the major issues that need attention in the order they are presented in the manuscript:

1) Two of the highlights are not supported by the data presented in the manuscript:
    - The opioid burden is rising in Bihar -- Evidence to support this is anecdotal and insufficiently detailed to include this point as a highlight.
    - Effective and evidence-based care can be delivered by integrating HCV care into OST-center – nothing is presented about care delivery; instead, the manuscript suggests that the authors feel that there an opportunity to do. This argument is presented in the Discussion as is appropriate, but it is not a highlight of the study.
2) The authors use the term “injection drug user(s)” and the abbreviation IDU. It would be better to use the term “people who use drugs” and the abbreviation “PWID”, which describe the medical condition of these individuals rather than define them as human beings. PWID should be used throughout the manuscript. Also, the term “addict” is pejorative and is to be avoided. The individuals in the study have been diagnosed with and are being treated for opioid use disorder (OUD) and should be described as such.
3) The authors write, “Bihar, one of the eastern states in India, has the least prevalence of opioid use (0.1%) compared to the national average of 0.7% [8]. Citation is about cardiovascular disease, not opioid use. Nothing in the cited article is relevant to this point the authors wish to make. Either identify a correct reference or delete.
4) The authors report that OST is initiated by a medical officer if an individual meets a set of criteria, one of which includes the individuals being “[c]urrent IDUs”. Does this mean that people with OUD but who are injecting drugs are excluded from treatment? If those not injecting are eligible for treatment, then the authors need to make clear that their sample is restricted to those patients who report a history of injection. Also, the authors report the age for eligibility as >18; does this mean that those 18 years of age are excluded. This has implications for how subsequent data are reported. In Table 1 the youngest age category is ≤20; in Figure 1 it is <20. Which is correct? And if there is a lower age cut off (either 18 or 19), then category should best be reported as 1x-20 or 1x-19, and if those 18 years of age are excluded, then the youngest category would be either “19” or “19-20”. Furthermore, the authors need to comment on the fact that nearly 20% of their sample fall into a category that covers a very few number of ages: some or all of those aged 18-20. It is quite

unusual for a treatment-based sample to have such a high percentage of individuals who are so young.

5) In the Methods, the authors report that age was expressed as means and standard deviation (SD). This is not strictly speaking correct. This is only true for the presentation of the data in the top row of Table 1 and (I believe) in Table 2. It is presented subsequently in Table 1 and in Figure 1 as a categorical variable. This needs clarification.
6) The authors describe their study design as cross-sectional, but they are including longitudinal data beyond the baseline when they introduce the results on mortality in their sample. The authors can either revise the study methods to mention that follow-up mortality data were obtained or they can delete this sentence since the difference in mortality as a function of HCV seropositivity was not found to be significant.
7) In the text, the authors report that "There was a rising prevalence of hepatitis C with age (p-trend= 0.811)." But this trend does not appear to be significant and since the regression is linear, the authors should report the r (or r^2) statistic as well as the p-value. Also, regarding Figure 1, the curve depicting HCV prevalence should not begin at the origin since the first real data point is for individuals aged <20, but it could be for those aged 18-19, 18-20, or just those aged 19. Please be sure that reporting of this youngest age group is consistent in the Methods, Results, Table 1, and Figure 2.
8) In the Discussion, the authors state, "We also found that younger patients (<35 years of age)…had a higher burden of HCV than their counterparts." The statistical analysis in the model presented in Table 2 does not support this conclusion; instead, age does not appear to be statistically associated with prevalence. In this part of the discussion, the authors could comment that the small sample size might have had insufficient power to identify trends associated with age.
9) In the Discussion, the authors opine that, "OST attendees or IDUs seeking services from any de-addition center differs from their counterparts; Such as they are more responsible, more educated, have more family and friend support and higher socio-economic status, and have less needle sharing behaviour etc." No data are presented to support the suggested differences. The authors' speculations may represent their prejudices more than they fairly describe differences between OTP patients and PWID not in treatment. If the authors want to keep this sentence, the directionality of differences between patients and non-patients should be directionless. For example, they could state that the two groups COULD differ in education attainment, social support, and behavioral risks.
10) In the Discussion, the authors comment that, "…we have also observed a very high HCV sero-prevalence among HIV-positive patients: 58.3% (26.7% - 84.3%). Such finding is expected as HIV significantly alters the pathological course of HCV [11, 12]. HCV patients co-infected with HIV show increased viral load, hepatic decompensation and mortality from the complications of end-stage hepatic diseases compared to HCV mono-infected individuals [13-16]." All of this is irrelevant, especially since the difference in HIV status as a function of HCV status was not significant (p=0.115 as reported in the text and in Table 1). The study is not assessing the course of HCV-related liver disease. The diagnostic test does not even reveal if a patient who tested seropositive is actively infected or has cleared the virus. Therefore, there is no need for any discussion of disease progression or mortality. It is enough to state that the finding of higher HCV prevalence in those co-infected with HIV is

consistent with global data and that HCV infection frequently precedes HIV infection because HCV is more infectious.

11) As noted earlier, the curves presenting the prevalence of HCV and confidence intervals in Figures 1 and 2 should not begin at the origin but at the first data point.

Below are the typographical, grammatical, and semantical errors that need to be addressed, again listed by the appearance in the pdf of the manuscript.

Background and Rationale

1) In the first paragraph, the text reads, "Thus at a given point in time, ~55 lakh patients are HCV seropositive and over the period of next 20 to 30 years ~15 lakh of these will develop chronic hepatic illnesses…" What is "lakh"? I assume it is the standard epidemiological denominator of 100,000. It would be better to use the numerical formulation since lakh seems to be specific to Indian English (according to OxfordLanguages).
2) The authors write, "Opioid substitution therapy (OST) canters/clinics have been developed to support opioid addicts (IDUs) to quit…" It would be more consistent with currently agreed upon terminology to revise this to read, "Opioid substitution therapy (OST) centers/clinics have been developed over the course of the last three decades to treat individuals with opioid use disorder (OUD) and have now escalated…"

Methods

3) The authors write, "The OST clinic started at the RMRIMS, Patna since January 2014 as a part of NACO (funded by Ministry of Health & Family Welfare, India) to reduce the transmission of HIV among IDUs." This sentence should read, "The OST clinic was started at the RMRIMS, Patna in January 2014 as a part of NACO's mission (funded by Ministry of Health & Family Welfare, India) to reduce the transmission of HIV among PWID."

Results

4) There is an opening parenthesis missing in the 2nd sentence of the first paragraph.
5) The authors write, "Participants with HCV positive status (compared to HCV negative status) were older (p-value= 0.732), unmarried or separated or divorced (p-value =0.122)…" Neither of these differences were significant and the text should reflect point; the authors can choose not to report these comparisons (as the data appear in Table 1) or they must state the differences were not statistically significant in the text.
6) Please define Avil and Penta using generic drug names for the international audience here and in Table 1. Also, please use a non-slang term for "smack" in Table 1. Does smack refer to heroin?
7) The authors write, "Participants with HCV positive status also had higher burden of HEV (p-value= 0.025) and HIV (p-value= 0.115)." This sentence should be corrected to read, "Participants with HCV positive status also had a higher burden of HEV (p-value= 0.025) but not of HIV (p-value= 0.115)."
8) In presenting the data in Table 1, since statistical testing is a comparison by HCV status, it would make more sense to have the totals column to the left, then two groups by HCV status before reporting the results of the statistical test.
9) In Table 1, the row heading should read "Total number of drugs use, median (IQR)".
10) The authors write, "There was a rising prevalence of hepatitis C with number of years of injection use (p- trend= 0.256)." The trend is not statistically significant. The text should

explicitly mention that the increase in prevalence was not significantly associated with number of years injecting.

Discussion

11) The parenthetical phrase "(who are addicted to injectable drugs)" should be deleted.
12) The authors write, "The observed HCV prevalence among OST is little bit lesser than the prevalence reported in the meta-analyses by Goel et al…" This should be revised to read, "The observed HCV prevalence 34.7% (95% CI: 28.3%-41.5%) among OST is somewhat less than the prevalence reported in the meta-analyses by Goel et al…"
13) In the sentence that begins: Nevertheless, our finding suggests that every third patient…" "demand" should be revised to "demands".
14) Substantial editing is needed for the sentences that read:

    Several clinical, social and behavioural factors drive the higher prevalence of HCV among IDUs. These are: many IDUs are younger, homeless and live alone in poverty; have a higher mental health problems; do not seek or cannot receive quality healthcare without assistance. Additionally, many have history of crime, so do not access any social services for fear of being discovered and returning to prison. Appropriate context-specific interventions targeting this high risk population should be explored.

    I suggest:

    Several clinical, social and behavioural factors drive the higher prevalence of HCV among IDUs. Many PWID are young [no need to use the comparative], homeless and live alone in poverty. They may have a more mental health problems, and, as a consequence do not seek or cannot receive quality healthcare without assistance. Many may have history of crime, and so do not access social services for fear of being discovered and returning to prison. Appropriate context-specific interventions targeting this high-risk population should be explored.
15) The authors write, "The higher HCV sero-prevalence in our study suggests a worsening of HCV burden among IDUs very similar to studies conducted in other settings." This sentence does not make sense to me. The HCV prevalence is consistent other studies of PWID; it is not higher. No evidence is presented that the burden is increasing/worsening over time. I suggest either completely revising this sentence or deleting it.
16) The authors write, "Similarly, HCV sero-prevalence was…higher among HIV positive patients. The difference as reported in Table 1 was not significant, so this point should be deleted from the sentence.
17) In point (e) in the paragraph on public health significance, "HIV care across car continuum" should be revised to read "HIV care across the continuum".
18) The References need to be carefully reviewed for format, capitalization, accuracy. Any citation of website or non-peer reviewed document that is not accessible with a DOI number should have a URL attached and a date that the URL was last accessed. For example, citation 5, "[5] Centers for disease control and prevention. Hepatitis c faqs for the public. 2022". This should be revised to state the publishing organization the US centers for disease control and prevention. (I understand that the British convention is to capitalize only the first word in an organization or official title, but for ease of understanding, I think the letter identifying a specific hepatitis visus should always be capitalized (or is it

capitalised?)) The title should read Hepatitis C FAQs, and URL and last access date needs to be added.
