# Supplementary material for: Hepatitis C virus seroprevalence among patients enrolled at the opioid substitution therapy center in Bihar: A cross-sectional study
Source: PLoS One. 2023 Jun 15;18(6):e0287333. doi: 10.1371/journal.pone.0287333 (PMC10270565; doi:10.1371/journal.pone.0287333)
Supplement: Attachment — Submitted filename: Response to Reviewers.docx [file pone.0287333.s003.docx]

Name of the manuscript: The burden of Hepatitis-C virus infection among patients enrolled at the opioid substitution therapy center in Bihar: A cross-sectional study

Authors: Ashish Kumar, Hemant Mahajan, Sanjay Chaturvedi, Ashok Kumar, Shiril Kumar, Ganesh Chandra Sahoo, Vidya Nand Rabi Das, and Krishna Pandey;

Corresponding author: Dr. Krishna Pandey; Director & Scientist-G, Rajendra Memorial Research Institute of Medical Sciences, Agamkuan, Patna- 800007, Bihar, India

drkrishnpandey@yahoo.com; 091-0612-2631565

**RESPONSE TO EDITOR’S AND REVIEWERS’ COMMENTS**

**Editor’s comments**

Please include the following items when submitting your revised manuscript:

- A rebuttal letter that responds to each point raised by the academic editor and reviewer(s). You should upload this letter as a separate file labelled 'Response to Reviewers'

Response: Appropriate rebuttal letter named 'Response to Reviewers' has been drafted and submitted at the designated PlosOne submission portal.

- A marked-up copy of your manuscript that highlights changes made to the original version. You should upload this as a separate file labelled 'Revised Manuscript with Track Changes'.

Response: A revised document with all the suggested modifications highlighted in Yellow has been drafted, labelled as 'Revised Manuscript with Track Changes, and uploaded at the PlosOne submission portal.

- An unmarked version of your revised paper without tracked changes. You should upload this as a separate file labelled 'Manuscript'.

Response: A revised document with all the suggested modifications has been drafted, labelled as ‘Manuscript’, and uploaded at the PlosOne submission portal.

**Journal Requirements**

- Please review your reference list to ensure that it is complete and correct. If you have cited papers that have been retracted, please include the rationale for doing so in the manuscript text, or remove these references and replace them with relevant current references. Any changes to the reference list should be mentioned in the rebuttal letter that accompanies your revised manuscript. If you need to cite a retracted article, indicate the article’s retracted status in the References list and also include a citation and full reference for the retraction notice.

Response: We have reviewed the reference list. It is complete and correct. We have not cited any paper that has been retracted. We have added two new references (Ref 5 and Ref 11) in the reference list and highlighted in yellow.

**Reviewers’ comments and response**

**Reviewer #2**

**Specific comments for ‘Introduction’ section**

Comment 1. I recommend changing the title to “Hepatitis C virus seroprevalence…” SInce about 30% of seropositive individual clear the virus, testing for antibodies does not accurately reflect the burden of infection but rather just evidence of past infection that could have become chronic.

Response: We appreciate reviewer’s suggestion and changed the title to “Hepatitis C virus seroprevalence among patients enrolled at the opioid substitution therapy center in Bihar: A cross-sectional study”

Comment 2. Intro paragraph 1, comment on prompt treatment for HCV: No, prompt treatment is not necessary as time between infection and life-threatening manifestations of end-stage disease in measured in decades.

Response: We believe that the presence of HCV acute infection warrants expedited linkage to care with a specialist engaged in the management of hepatitis patients. The spontaneous clearance of HCV infection generally occurs in patients treated within 6 months of infection and significantly reduces the chances of life-threatening manifestations of HCV infection. (Ref 5. Guss D et al. Diagnosis and Management of Hepatitis C Infection in Primary Care Settings. Journal of general internal medicine 2018.)

Comment 3. Paragraph 2 “Health care related procedures, injection drug use, and blood transfusions contribute to the spread of the HCV virus” delete “virus”

Response: We have deleted the term virus after HCV.

Comment 4. Revise “HCV transmission has reduced significantly through blood transfusion” to read, “HCV transmission through blood transfusion has been significantly reduced….”

Response: We have revised the statement “HCV transmission has reduced significantly through blood transfusion” to “HCV transmission through blood transfusion has been significantly reduced” in the manuscript.

Comment 5. Paragraph 3 text that reads “Traditionally, it has been seen that ~15% PWID have HIV/AIDS…” should be revised to read “Traditionally, it has been seen that ~15% of PWID has HIV/AIDS…”

Response: We have revised the statement “Traditionally, it has been seen that ~15% PWID have HIV/AIDS…” to “Traditionally, it has been seen that ~15% of PWID has HIV/AIDS…” in the main manuscript.

Comment 6. Paragraph 4, last sentence currently reads, “…it is important to have a good quality epidemiological data with reasonable accuracy.” Revise to read, ““It is important to have reasonably accurate epidemiological data.”

Response: We have revised the statement ““…it is important to have a good quality epidemiological data with reasonable accuracy” to “It is important to have reasonably accurate epidemiological data …” in the main manuscript.

**Specific comments for ‘Methods’ Section**

Comment 1. Study population, 1st paragraph – text reads “Whenever a patient visits OST canter (to seek services)”, fix typo “canter”.

Response: We have fixed the typo “canter”.

Comment 2. Study population, 1st paragraph – text reads “All patients at baseline also undergo for HIV, HBV antigen, and HCV antibody tests,” Revise the read “All patients at baseline also undergo testing to detect HIV, HBV antigen, and HCV antibody

Response: We have revised the statement “All patients at baseline also undergo for HIV, HBV antigen, and HCV antibody tests,” to “All patients at baseline also undergo testing to detect HIV, HBV antigen, and HCV antibody.

**Specific comments for ‘Results’ section**

Comment 1. First paragraph – In the sentence, “The HCV seropositivity prevalence was 28% [95% CI: 22.7% - 33.8%)].” Delete the bracket at the end.

Response: We have deleted the bracket.

Comment 2. Generic and street drug names should not be capitalized in the text; okay to capitalize a row name in the tables.

Response: As suggested, we have made the appropriate changes in the manuscript.

Comment 3. Age is analyzed in two ways – as means via unpaired t-test and as a categorical variable. In the second paragraph of the Results, it appears that the analysis of age used the t-test. It would clarify things if this were indicated by a footnote in Table1.

Response: As suggested, we have made the appropriate changes in the manuscript. We have added clarifying footnote in Table 1.

Comment 4. Third paragraph – Although the test for trend is significant, there really is not rising prevalence. The sentence that read “There was a rising prevalence of HCV seropositivity with age (p-trend= 0.025).” should be deleted. The next sentence better describes the situation. Prevalence was lower in the two youngest age groups compared to the four older ones, which were quite similar.

Response: We have made appropriate changes in the manuscript.

Comment 5. Third paragraph – Fix typo in next sentence to read: Patients aged 25 years or less had..”

Response: We have made appropriate changes in the manuscript.

Comment 6. In the fourth paragraph, the 2nd and 3rd sentences are inconsistent with each other. Prevalence is higher in each group with more years of injecting compared to the group with fewer years — a monotonic increase in prevalence. The sentence about a sharp increase among those injecting >2 years suggests that thereafter prevalence did not increase much after that first increase after 2 years injecting, but this is not what that the data show. Delete the sentence about the sharp increase.

Response: We have made appropriate changes in the manuscript.

Comment 7. In the fifth paragraph, fix typo to read, “Patients with education up to higher…”

Response: We have made appropriate changes in the manuscript.

Comment 8. The last sentence in the fifth paragraph about the association of longer time injecting and HCV seropositivity should be moved to the previous paragraph discussing the association between years of injection and HCV seroprevalence

Response: Thank you for the suggestion. The findings in the fourth paragraph are describing the unadjusted association between years of injection use and HCV prevalence. Whereas, the fifth paragraph is describing the association between years of injection and HCV seroprevalence after adjusting for the effect of sociodemographic and other clinical variables.

**Specific comments for ‘Discussion’ Section**

Comment 1. First paragraph – fifth sentence should be revised to read “We also found that those unemployed, less educated, and who were using injection drugs for longer period of time had a higher HCV seropositivity than their counterparts.”
Response: We have made appropriate changes in the manuscript.

Comment 2. Second paragraph – In the second sentence delete the mention of the confidence interval and the sentence can be rephrased to read “The observed HCV prevalence of 28% among OST patients in Bihar was significantly less than the 44.7% prevalence reported by Goel et al. in their systematic analysis of HCV in PWID across India.”

Response: We have made appropriate changes in the manuscript.

Comment 3. In suggesting reason for the lower prevalence, you should note that geographic differences may also play a role.

Response: As suggested we have added the required information (with appropriate citation) in the discussion section.

Comment 4. Second paragraph – Revise sentence to read “Nevertheless, our findings suggest that approximately 30% of patients….”

Response: We have made appropriate changes in the manuscript.

Comment 5. Second paragraph, last sentence – revise to read “Unfortunately, the available clinical records for the OST attendees in ICMR-RMRIMS do not provide any information about adverse exposures, prohibiting comparison with other studies.”

Response: We have made appropriate changes in the manuscript.

Comment 6. Public health significance – The first sentence is one very long run-on with five distinct parts. Each should be its own sentence.

Response: We have broken down the long sentence into four to five small sentences.

Comment 7. Strengths and limitations -- A major limitation is the very small sample size. The clinic enrolled, on average, fewer than 30 patients every year. The barriers to entry — daily or near daily visits, active injection, failure of non-medication treatment for OUD — made for not only a small but potentially very biased sample.

Response: We really appreciate reviewer’s suggestion. We have added the *limitation of small sample size* in the revised manuscript.

**Specific comments for ‘Conclusion’ Section**

Comment 1. Delete “a” from the 3rd sentence “…where a comprehensive HCV care…”

Response: We have made appropriate changes in the manuscript.

**Specific comments for ‘Figures’**

Comment 1. Both figures need to be revised. The current figures are not the correct way to present the data on HCV prevalence and age or years of injection, and the presentation actually complicates interpretation of the data. Since age and years of injection are presented as categorical variables, the HCV data should be represented by a bar with the confidence interval stretching above and below the bar. There is no way that prevalence and 95% CI should be shown as curves even if data were analyzed using Poisson regression. There is no need to present the data on the %age of study participants in each category (age and years of injection) as a bar; this information could be placed in text box above bar.

Response: As per reviewer’s suggestion, we have modified figures (1 & 2) and put the proportion of participants for ‘age group’ categories as well as ‘years of injection use’ categories in footnote for Figure 1 and 2.
